# First clinical study of a novel complete metal-free ceramic total knee replacement system

**DOI:** 10.1186/s13018-016-0352-7

**Published:** 2016-02-08

**Authors:** E. Meier, K. Gelse, K. Trieb, M. Pachowsky, F. F. Hennig, A. Mauerer

**Affiliations:** Department of Orthopaedic and Trauma Surgery, University Hospital Erlangen, Krankenhausstr. 12, 91054 Erlangen, Germany; Hospital Wels, Grieskirchnerstr. 42, A-4600 Wels, Austria

**Keywords:** Arthroplasty, Knee, TKA, Ceramic, Hypersensitivity

## Abstract

**Background:**

The aim of the study was to evaluate the safety and efficacy of a novel metal-free ceramic total knee replacement system.

**Methods:**

Thirty-eight primary total knee arthroplasties (TKAs) were performed on 34 patients using the metal-free BPK-S ceramic total knee replacement system with both the femoral and tibial components of an alumina/zirconia ceramic composite. The clinical outcome was evaluated pre- and postoperatively at 3 (*n* = 32 TKA) and 12 months (*n* = 32 TKA) using the Knee Society Score (KSS), the Oxford Knee Score and the EQ-5D. Safety analysis was performed by radiological examination and assessment of adverse events.

**Results:**

Postoperatively, the KSS, Oxford Knee Score and EQ-5D improved significantly at 3 and 12 months (*p* < 0.001). Non-progressive partial radiolucent lines were observed in six cases, but there was no osteolysis and no implant loosening. Induction or exacerbation of allergies did not occur during the follow-up.

**Conclusions:**

The metal-free BPK-S ceramic total knee replacement system proved to be a safe and clinically efficient alternative to metal implants in this short-term follow-up study.

## Background

One major factor limiting the implant survival in total knee arthroplasty (TKA) is aseptic loosening due to immune reactions resulting from the agglomeration of debris as a result of wear [1–3]. In order to reduce the amount of implant wear, bearing couples of different materials have been investigated for decades. Due to favourable tribological properties, a significant reduction in wear rates of ceramic bearings has been well demonstrated in vitro and in vivo [4–6]. Furthermore, the release of wear particles may also elicit allergenic reactions, which are observed with metal implants [7]. Indeed, the prevalence of metal hypersensitivity is approximately 10–15 %, with nickel sensitivity having the highest prevalence in the presence of a common cross-reactivity between nickel and cobalt [8]. Elevated cobalt and chromium serum ion levels were reported in patients after implantation of hip replacement devices [9]. Immunological metallic-specific responses to metal ions and metal debris are considered possible causes for postoperative pain, poor performance, osteolysis and early implant loosening [10, 11].

These data underline the rationale for an implant that is completely free from metal components. However, the brittleness and low tensile strength with the risk of material fractures are considerable problems for the use of ceramics in joint replacement. This is particularly true for the complex shape of TKA components. Indeed, material fractures of ceramic TKA components have been documented in some studies [12–14]. Majima et al. described the need for revision surgery in one of 110 cases because of breakage of the ceramic tibial tray [13]. Bergschmidt et al. observed four fractures of the ceramic femoral condyle at the time of the implantation procedure at the beginning of their study [12]. In another case report, a fracture of the patellar groove of the ceramic femoral component with a still intact cement mantle was ascribed to a traumatic event [14].

For this reason, the use of ceramic has been confined to the femoral TKA component so far. However, the development of novel ceramic composites with improved tensile strength and fracture toughness have also made it possible to use ceramic for the tibial component. The improved mechanical properties of the Biolox®delta ceramic composite were yielded by an alumina matrix containing uniformly distributed particles of tetragonal zirconium oxide.

The aim of this prospective, non-controlled single-arm, open-label, observational study was to investigate the efficacy and safety of the complete metal-free BPK-S Integration ceramic knee replacement system.

## Methods

### Study population

A total of 34 patients were enrolled in this study. This population included 38 TKAs (*n* = 38 TKA) with 21 (55.2 %) TKA in female and 17 (44.7 %) TKA in male patients. Surgery was performed on 17 (44.7 %) left and 21 (55.2 %) right knees. The mean age of the study population at inclusion was 66.87 ± 9.76 (46–83) years. The median height and weight of the patients were 172.5 (152.0–190.0) cm and 92.5 (61.0–156.0) kg, respectively. The average BMI of the patients was 31.4 ± 5.6 (22.7–47.8) kg/m^2^.

This study was performed as a prospective, non-controlled single-arm, open-label, observational study for the clinical and radiological evaluation of the metal-free BPK-S Integration total knee replacement system with femoral and tibial components of Biolox®delta ceramic composite and an ultra-high-molecular-weight polyethylene (UHMWPE) insert. The study was classified as evidence-based medicine (EBM) level 4. Inclusion criteria were the indications of primary TKA due to primary or posttraumatic osteoarthritis or rheumatoid arthritis, congenital or acquired knee joint defect or deformation, and a known history of metal hypersensitivity. Contraindications were severe damage to bone structures which could impede the stable fixation of the implant components, infection near the implantation site, cancer and renal dialysis. Patients with drug abuse, mental diseases or lack of cooperation were excluded from the study. The study was approved by the institutional ethics committee of the medical faculty of the University Erlangen-Nuremberg, and all patients gave their written, informed consent.

### Total knee replacement system

**Fig. 1:**
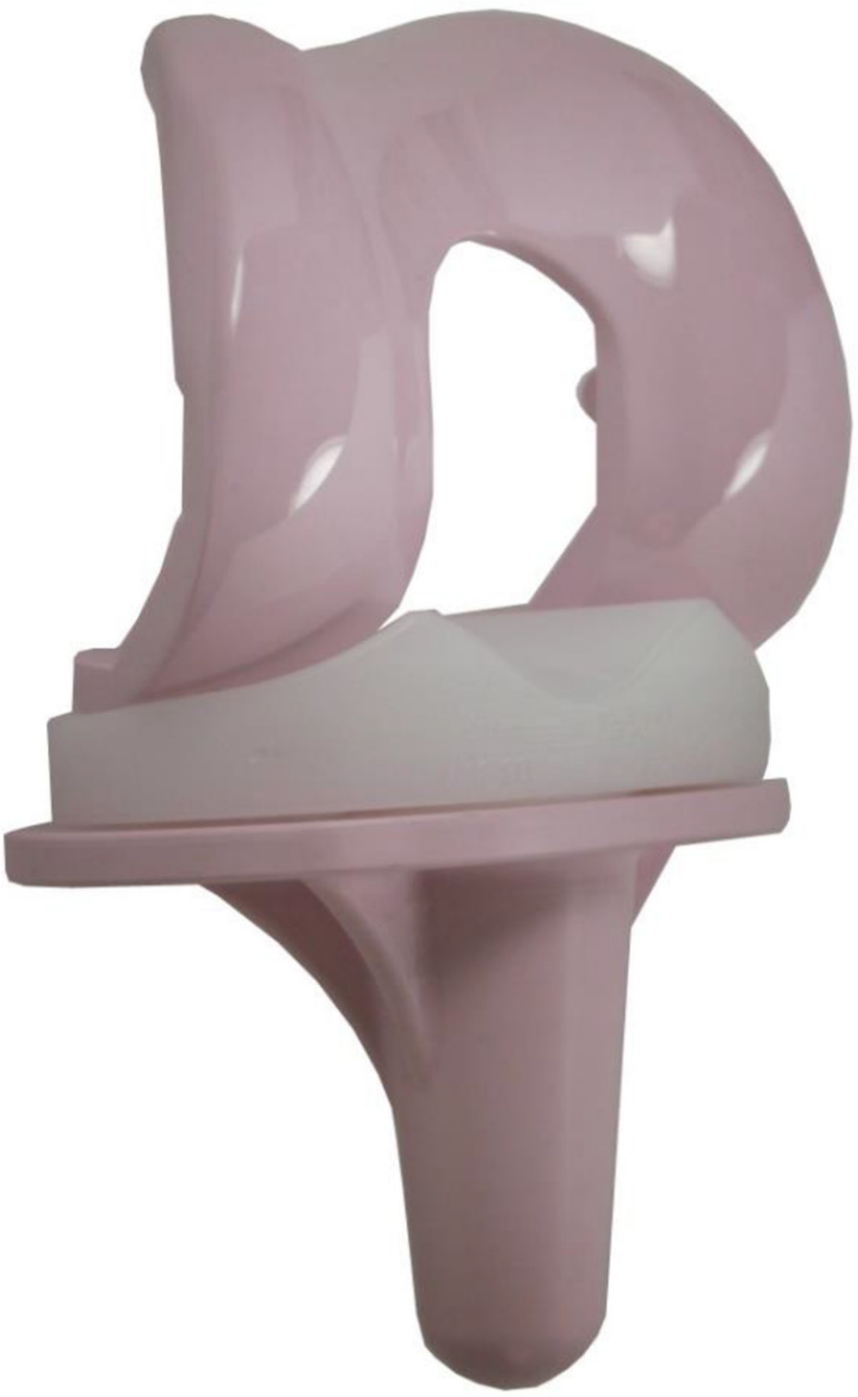
BPK-S Integration total knee replacement system with femoral and tibial component of Biolox® ceramic composite (Peter Brehm, Germany)

The BPK-S Integration is a non-constrained primary total knee replacement device (Peter Brehm GmbH, Weisendorf, Germany) with both the bicondylar femoral element and the tibial component of alumina/zirconia ceramic composite (Biolox®delta; CeramTec AG, Plochingen, Germany) (Fig. 1). The rotating or fixed-bearing insert is made of UHMWPE. The device can be combined with a patella replacement made of UHMWPE. The geometrical design of the ceramic BPK-S Integration is identical to the clinically established cobalt-chromium BPK-S Integration by the same manufacturer. The mechanical and clinical efficacy and safety of the conventional cobalt-chromium BPK-S TKA has been proven in recent studies [15, 16].

Prior to this clinical study, the metal-free ceramic BPK-S tibia component underwent extensive mechanical tests under experimental conditions, which were performed according to the standards ISO 14879-1:2000(E) and ASTM F1800-07. All of the tested BPK-S ceramic tibia components (size “3” *n* = 7; size “6” *n* = 2) withstood the alternating load test with ten million cycles of sinusoidal dynamic stress (10 Hz) with 5300 N topload and 530 N underload. The subsequent post-fatigue burst strength test revealed the maximum strength against fracture of the same specimen. The burst strength until failure amounted to 11.8 ± 1.5 kN for size “3” tibial components or 13.6 ± 2.0 kN for size “6” tibial components, respectively. These experiments demonstrated large reserves in the mechanical strength of the ceramic tibia components, which exceeded by far the required safety norms (DIN EN ISO 21536:2009).

### Intra- and perioperative management

Long-leg standing radiographs were the basis for preoperative planning using a digital surgical planning software (MediCAD, HECTEC GmbH, Landshut, Germany). The alignment was adjusted to the mechanical axis with the femoral and tibial resection lines perpendicular to the mechanical axis of the femur and tibia according to Insall et al. [17]. The pre-defined implant slope was 6°. The cutting levels were defined by the digital planning software. All TKAs were implanted by conventional surgical methods using the standard instruments from the manufacturer intended for implantation of the established cobalt-chromium BPK-S with the exception of a single-use plastic-coated peg of the applicator for the femoral component and a modified applicator for the tibial component. These modified applicators prevented the metal instruments from having any contact with the ceramic components. The femoral and tibial ceramic components were fixed to the bone in a one-stage technique with polymethyl-methacrylate (PMMA) bone cement (Stryker Simplex; Duisburg, Germany). The postoperative mobilization was performed according to the current standards. On the first day after surgery, the patients began exercising their knee joint in an unlimited range of motion under the supervision of a physiotherapist and use of a continuous passive motion (CPM) device. They were allowed a partial weight-bearing of 20 kg for the first 2 weeks followed by the transition to full weight-bearing after wound healing.

### Radiological evaluation

The radiological evaluation was performed by standard anterior-posterior and lateral X-ray imaging preoperatively, directly postoperatively (day of surgery), as well as 3 and 12 months after surgery. Long-leg standing radiographs were done preoperatively and postoperatively at the time of discharge. The images were evaluated for tibial and femoral implant positioning and alignment, periprosthetic fractures, radiolucent lines and osteolysis by three independent observers according to the Knee Society roentgenographic evaluation [18].

### Clinical evaluation

The efficacy of the TKA procedure was performed preoperatively (baseline), as well as at 3 and at 12 months postoperatively using the Knee Society Score (KSS), the Oxford Knee Score and the EQ-5D plus VAS. Safety variables included early and late TKA revisions, radiological signs of functional or structural alterations (loosening, wear, dislocation, deformation and damage), medical device incidents, and intra-/peri-/or postoperative complications throughout the entire study period.

### Statistical analysis

All data were analysed using the SPSS statistical package (SPSS Inc., Chicago, USA). The data of KSS, Oxford Score and EQ-5D are presented as mean ± SD (95 % CI) or by boxplot analysis showing minimum, 25 % quartile; median, 75 % quartile; and maximum values. The efficacy of the procedure with comparison of the outcome at the different time points was evaluated using the two-sided Wilcoxon sign-rank test. *p* values lower than 0.05 were considered significant.

## Results

A total of 34 patients were enrolled in the study (*n* = 38 TKA). Out of these, 28 patients (*n* = 32 TKA) completed the 3- and 12-month follow-up examination. Among the drop-outs during the study, five patients withdrew their consent for personal reasons, and one patient dropped out due to poor health status (stroke). There was no case of discontinuation related to the TKA device.

**Fig. 2:**
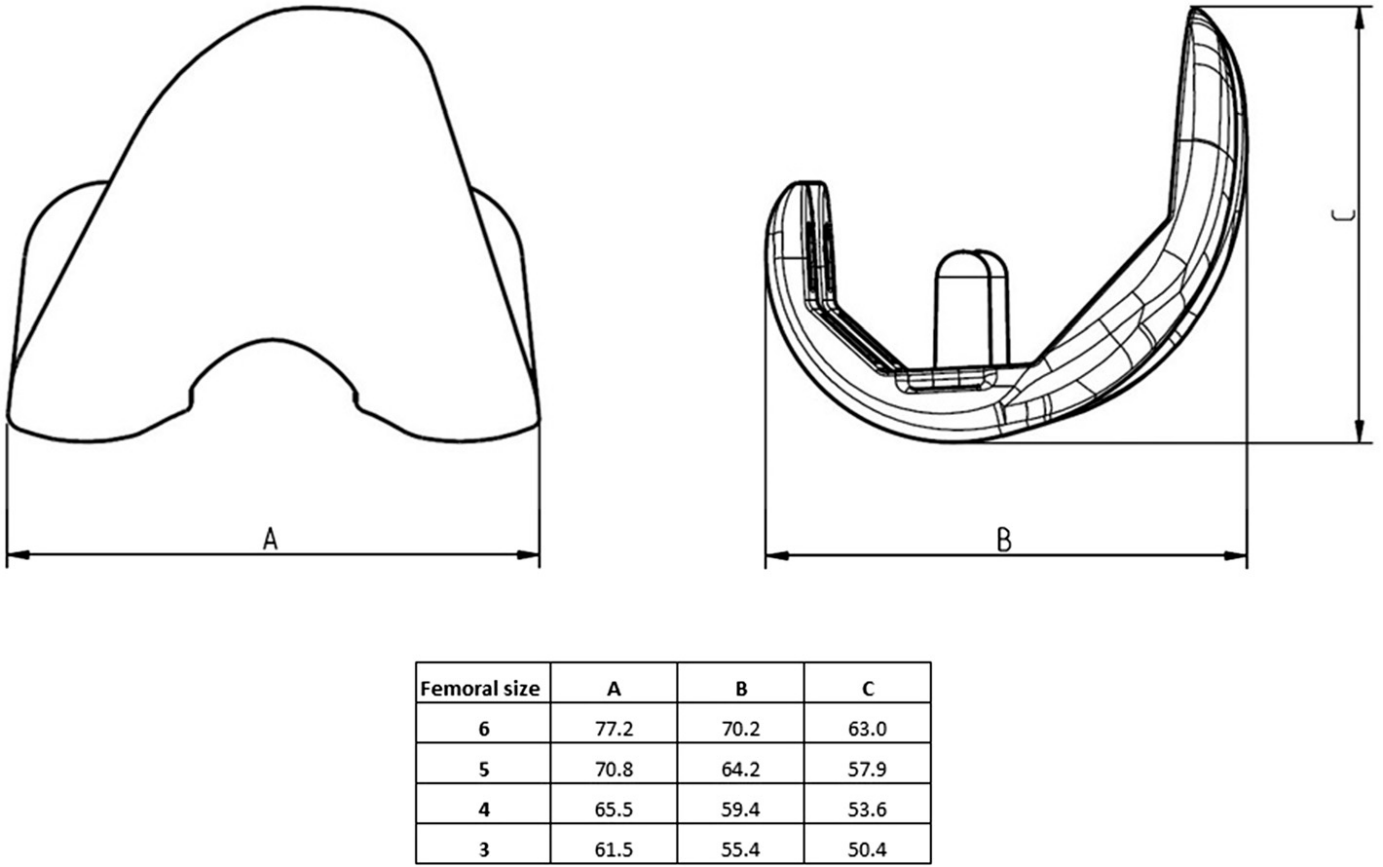
Size specifications of the femoral component of the BPK-S Integration total knee replacement system

**Fig. 3:**
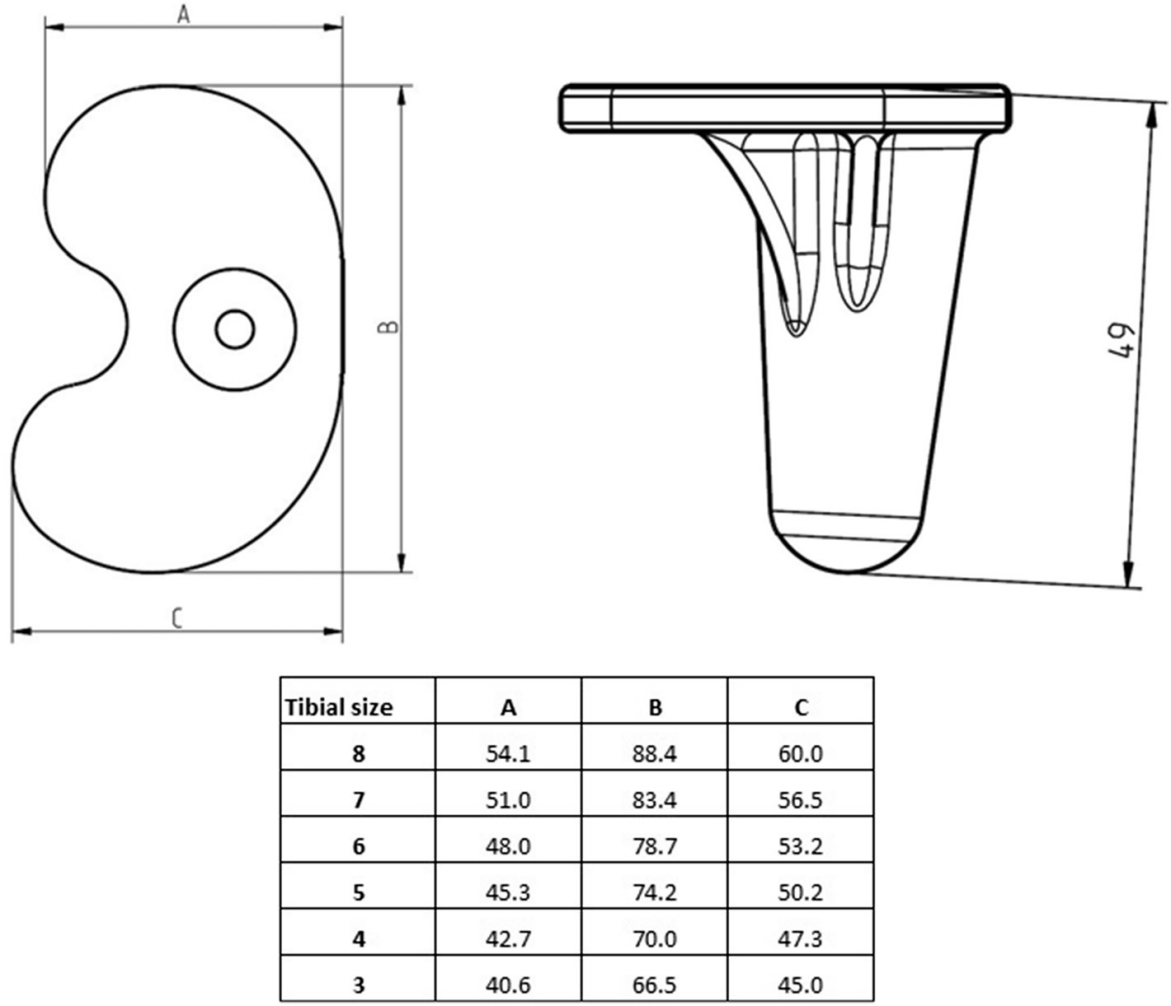
Size specifications of the tibial component of the BPK-S Integration total knee replacement system

Mean duration surgery time (*n* = 38) was 70.21 (SD ± 9.97) minutes. The size of the implants ranged from “4” to “6” for the femoral component, “4” to ”6” for the tibial component and “7” to “13” for the insert (Figs. 2 and 3). Patella replacement was not carried out on any patient in this study. There were no technical problems during the implantation. Intraoperative implant failure or problems related to the cement fixation of the ceramic components did not occur in any of the patients.

Postoperative radiographs demonstrated a mean anatomical femoro-tibial valgus of 6.18° ± 1.5° (range 4°–9°). The mean mechanical lateral distal femoral angle (mLDFA) was 89.7° ± 1.5° (range 87°–92°), which adequately corresponds to the preoperative planning (aberrance <3° in all cases).

### Efficacy outcome

**Fig. 4:**
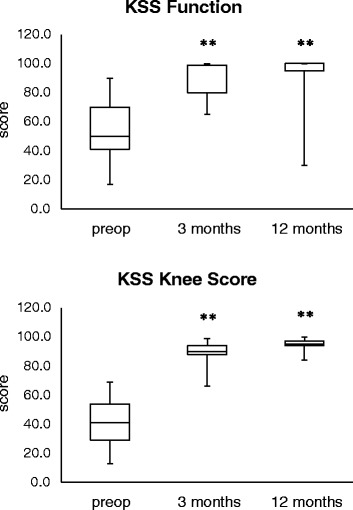
Boxplot analysis of the KSS score values (functional score and knee score preoperatively as well as at 3 and 12 months postoperatively (**p < 0.01 vs. preoperative)

The KSS (total score, subscore knee, subscore function), Oxford Knee Score and EQ-5D plus VAS improved significantly (*p* < 0.05) from the preoperative evaluation to the postoperative evaluations at 3 and 12 months. The “total” KSS score increased from a mean of 92.45 (SD 30.59, *n* = 38) at baseline (preoperative evaluation) to a mean of 174.22 (SD 15.61, *n* = 32) at 3-month follow-up and to a mean of 189.13 (SD 15.61, *n* = 32) at 12-month follow-up. The change from baseline was statistically significant (*p* < 0.0001) for both points in time (Fig. 4). The KSS “knee score” improved from a mean of 38.84 (SD 15.75, *n* = 38) at baseline (preoperative evaluation) to a mean of 88.44 (SD 9.09, *n* = 32) at 3-month follow-up and to a mean of 93.66 (SD 7.41, *n* = 32) at 12-month follow-up. The change from baseline was statistically significant (*p* < 0.0001) for both points in time (Fig. 4). The KSS “functional score” increased from a mean of 53.61 (SD 18.45, *n* = 38) at baseline (preoperative evaluation) to a mean of 85.78 (SD 9.51, *n* = 32) at 3-month follow-up and to a mean of 95.47 (SD 12.01, *n* = 32) at 12-month follow-up. The change from baseline was statistically significant (*p* < 0.0001) for both points in time (Fig. 4).

**Fig. 5:**
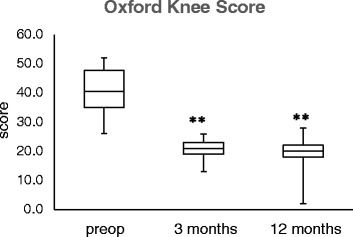
Boxplot analysis of the Oxford Knee Score preoperatively as well as at 3 and 12 months postoperatively (**p < 0.01 vs. preoperative)

The Oxford Knee Score decreased (clinically improved) from a mean of 40.84 (SD 7.40, *n* = 38) at baseline (preoperative evaluation) to a mean of 20.84 (SD 3.24, *n* = 32) at 3-month follow-up and 20.63 (SD 3.85, *n* = 32) at 12-month follow-up (Fig. 5). The change from baseline was significant (*p* < 0.0001) for both points in time.

**Fig. 6:**
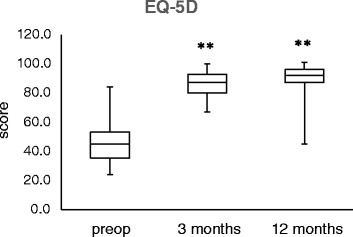
Boxplot analysis of the EQ-5D score values preoperatively as well as at 3 and 12 months postoperatively (**p < 0.01 vs. preoperative)

The EQ-5D increased from a mean of 46.24 (SD 14.13, *n* = 38) at baseline (preoperative evaluation) to a mean of 85.25 (SD 8.33, *n* = 32) at 3-month follow-up and 89.66 (SD 10.69, *n* = 32) at 12-month follow-up (Fig. 6). The change from baseline was significant (*p* < 0.0001) for all the points in time. The VAS improved from a mean of 33.24 (SD 14.88, *n* = 38) at baseline (preoperative evaluation) to a mean of 77.66 (SD 8.42, *n* = 32) at 3-month follow-up and 82.19 (SD 12.11, *n* = 32) at 12-month follow-up (Fig. 6). The change from baseline was significant (*p* < 0.0001) for both points in time.

### Safety outcome

**Fig. 7:**
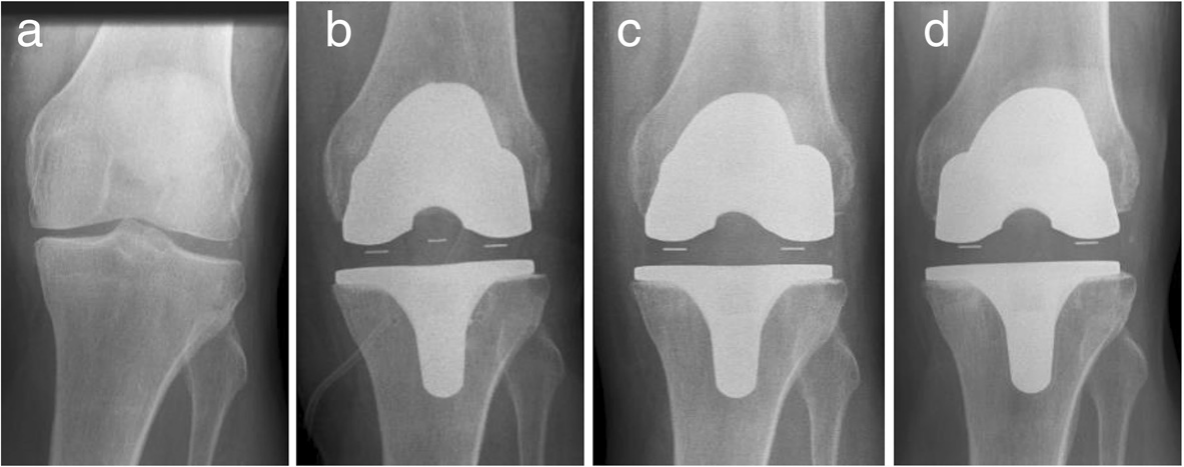
Radiographs of one patient preoperatively (**a**), postoperatively (day of surgery) (**b**), 3 months (**c**) and 12 months (**d**) after surgery

There were no failures of the implant during surgery or the reported follow-up period. There was no case of intraoperative nor postoperative periprosthetic bone fractures. Non-progressive radiolucent lines were seen in one femoral component (zone 1) and five tibial components (*n* = 2 in zone 1 (lateral view); *n* = 1 in zone 1 (a.p. view); *n* = 1 in zone 1 + 2 (a.p. view); *n* = 1 in zone 3 + 4 (a.p. view)). All radiolucent lines were measured less than 1 mm in width and were observed between the bone and cement. All radiolucent lines were observed at the 3-month follow-up and showed no further progression at the 12-month follow-up. There was no loosening of the TKA device in any of the patients. The positioning of the implants remained correct throughout the follow-up period (Fig. 7).

There were a total of three complications that all occurred during the follow-up period. In one patient, a surgical superficial wound revision (without revision of the implant) was necessary, which subsequently healed without any further complications. One patient had an out-of-hospital stroke with a persisting hemiparesis 1 month after TKA implantation. A causality of the stroke event with the implanted TKA was not apparent. One patient developed a deep vein thrombosis 3 months after TKA implantation. The thrombosis was resolved without reoccurrence by thrombolytic and subsequent antithrombotic medication.

## Discussion

This is the first study investigating the clinical outcome and safety of a completely metal-free total knee replacement system with both femoral and tibial components made out of ceramic material. The complex biomechanical loads with considerable bending and torsional forces have challenged the use of ceramic for the tibia component and have limited the use of ceramic for the femoral component so far [12, 19]. The development of a novel alumina/zirconia ceramic composite has made it also possible to build the tibial component out of ceramic.

In the limited follow-up of 12 months of this study, we could not detect any device-related complications. There was no implant breakage or aseptic loosening in any of the patients of this present study. These encouraging results concerning implant safety may be a result of the unique Biolox®delta ceramic material, which is a composite matrix material containing aluminium oxide (AL_2_O_3_) and zirconium oxide (ZrO_2_) with improved mechanical characteristics in terms of strength and resistance [20]. Furthermore, careful intraoperative handling was performed with strict avoidance of any contact of the ceramic components by metal instruments. For this purpose, unique applicator devices with single-use plastic-coated pegs were developed.

Radiological examination revealed radiolucent lines around one femoral component and five tibial components, which were all observed between the bone and cement. The frequency of radiolucent lines is comparable to those in other studies using metal TKA devices and may be ascribed to stress-shielding phenomena or poor cement penetration due to sclerotic bone [21]. The radiolucent lines were not progressive, and there was no aseptic loosening of the ceramic TKA components in this study. Due to the lack of any loosening event of a ceramic component, TKA survival rates were not calculated in the present study but will be addressed in the ongoing course of study.

Finally, none of the patients developed an allergic reaction to the implant or exacerbation of existing hypersensitivities. This underlines the rationale of a complete metal-free TKA for the use in patients with known hypersensitivities.

The analysis of the efficacy demonstrated that all patients significantly gained benefit from the implantation of the ceramic TKA by improved or restored knee function, mobility, quality of life and pain situation. In the present study, the KSS increased from 38.84 (knee score) or 53.61 (function score) at baseline to 93.7 or 95.5 at the 12-month follow-up, respectively. Thus, the functional outcome of the ceramic BPK-S Integration is comparable to established TKA devices. Recently, two large studies reported postoperative KSS (function score) values between 76.9 and 94.2 at the 12-month follow-up with the standard NexGen knee (Zimmer) or its high-flexion version [22, 23]. One study compared the clinical outcome of the AGC V2, Duracon and NexGen TKA devices and revealed KSS (knee score) values of 90.0, 79.9 and 86.0 and KSS (function score) values of 97.7, 90.5 and 96.2, respectively [24].

Thus, with respect to the clinical performance, the BPK-S ceramic TKA device meets the functional performance of established primary standard metal TKA systems that are currently in clinical use. Furthermore, the implantation of the BPK-S ceramic implant does not require any differential inclusion nor exclusion criteria. For the surgeon, the switchover to the ceramic TKA system appears to be completely unproblematic, since the surgical technique and target alignment are comparable to the current metal component system.

Some limitations of this study have to be considered when interpreting the data. First, it was a non-randomized single-arm study. Follow-up evaluations were limited to 3 and 12 months postoperatively so far and, thus, do not allow the evaluation of, e.g. polyethylene wear. Nevertheless, this pilot study could exclude severe complications related to the ceramic TKA device that may predominantly occur during surgery or within the first postoperative months. Revision analyses of TKA surgery demonstrated that about 19 % all revisions had to be done in less than 12 months after the primary procedure [25]. However, longer follow-up periods will be required to draw final conclusions concerning the survival rate and polyethylene wear of the ceramic TKA and may be able to reveal the potential advantages of the ceramic TKS systems.

## Conclusions

This is the first clinical study on a completely metal-free TKA device with both the femoral and tibial component made out of Biolox®delta ceramic. The ceramic BPK-S Integration proved to be safe with no implant failure nor loosening in the limited follow-up period of 12 months. Thus, the ceramic implant may represent a promising alternative in particular for patients with a known hypersensitivity to metal. Long-term studies will have to approve the favourable short-term clinical outcome as well as implant survival rates.

### Ethical approval

All procedures performed in this study were in accordance with the ethical standards of the institutional and/or national research committee and with the 1964 Helsinki Declaration and its later amendments or comparable ethical standards. The study was approved by the institutional ethics committee of the medical faculty of the University Erlangen-Nuremberg.

### Informed consent

Informed consent was obtained from all individual participants included in the study.
